# Integrating roots into a whole plant network of flowering time genes in *Arabidopsis thaliana*

**DOI:** 10.1038/srep29042

**Published:** 2016-06-29

**Authors:** Frédéric Bouché, Maria D’Aloia, Pierre Tocquin, Guillaume Lobet, Nathalie Detry, Claire Périlleux

**Affiliations:** 1 InBioS, PhytoSYSTEMS, Laboratory of Plant Physiology, University of Liège, Quartier Vallée 1 Sart Tilman Campus, Chemin de la Vallée no. 4, B-4000 Liège, Belgium

## Abstract

Molecular data concerning the involvement of roots in the genetic pathways regulating floral transition are lacking. In this study, we performed global analyses of the root transcriptome in Arabidopsis in order to identify flowering time genes that are expressed in the roots and genes that are differentially expressed in the roots during the induction of flowering. Data mining of public microarray experiments uncovered that about 200 genes whose mutations are reported to alter flowering time are expressed in the roots (*i.e.* were detected in more than 50% of the microarrays). However, only a few flowering integrator genes passed the analysis cutoff. Comparison of root transcriptome in short days and during synchronized induction of flowering by a single 22-h long day revealed that 595 genes were differentially expressed. Enrichment analyses of differentially expressed genes in root tissues, gene ontology categories, and cis-regulatory elements converged towards sugar signaling. We concluded that roots are integrated in systemic signaling, whereby carbon supply coordinates growth at the whole plant level during the induction of flowering. This coordination could involve the root circadian clock and cytokinin biosynthesis as a feed forward loop towards the shoot.

Flowering is a crucial step in plant development that must be precisely timed to occur when external conditions are favourable for successful reproduction. Floral induction is therefore controlled by environmental and endogenous cues, whose inputs are integrated into finely-tuned regulatory gene networks. In *Arabidopsis thaliana*, genetic analyses unveiled several flowering pathways that mediate response to photoperiod, temperature, sugars, hormones, and plant aging^1^. These pathways are not restricted to the shoot apical meristem where flowers are initiated, but also involve the leaves supporting the fact that flowering is a systemic process, as shown previously at the physiological level. The clearest genetic evidence supporting this idea came from the photoperiodic pathway, in which a key component, the transcription factor CONSTANS (CO), is expressed rhythmically but is degraded in the dark^2^. Light must therefore coincide with CO synthesis to stabilize the protein and enable activation of its targets. This occurs during long days in the companion cells of phloem, where CO activates *FLOWERING LOCUS T* (*FT*). The FT protein then moves systemically and interacts with the transcription factor FD via 14-3-3 proteins in the shoot apical meristem. This complex activates genes that are responsible for the conversion of the vegetative shoot apical meristem into an inflorescence meristem and for the promotion of floral fate in lateral primordia.

The prominent role of the FT protein in the systemic signaling of flowering leads to questions concerning the role of side molecules that are co-transported from leaf sources in the phloem and the pleiotropic effects of these signals in different sinks. Sugar loading is the first step of mass-flow movement in phloem, hence carbohydrates might influence flowering signals delivery. Several reports, however, indicate that sugars themselves act as flowering signals at two sites in the plant. In the leaves, photosynthesis and activity of TREHALOSE-6-PHOSPHATE SYNTHASE 1 (TPS1), which catalyzes the formation of trehalose-6-phosphate (T6P), are required for the induction of the *FT* gene^3^,^4^. The plant thus integrates environmental signals such as photoperiod and physiological signals including sugar, to activate *FT* and induce flowering.

Interestingly, CO regulates the expression of *GRANULE-BOUND STARCH SYNTHASE* (*GBSS*), controlling the synthesis of amylose in starch granules, and could thereby mediate transitory starch composition to increase sugar mobilization during floral transition^5^. Using a starchless mutant, Corbesier *et al*.^6^ concluded that starch mobilization was critical for flowering in conditions that did not involve increased photosynthetic activity. Together, these results build evidence for sugar contribution to the florigenic signaling. In the shoot apex, sucrose content increases when Arabidopsis plants flower in response to a photosynthetic long day^3^,^6^ or eventually in non-inductive short days^7^. Sugars can induce the expression of flowering genes in the meristem independently of *FT*^4^. Beside sugars, the phloem sap of Arabidopsis is enriched in amino acids and hormones of the cytokinin family when flowering is induced by photoperiod^6^,^8^. Cytokinins can promote flowering by inducing the paralog of *FT*, *TWIN SISTER OF FT* (*TSF*), in the leaves and downstream flowering genes in the shoot apical meristem^9^.

If we can infer from the previous section that multiple flowering signals are transported in phloem, the signaling route appears to be unidirectional from leaves to the shoot apical meristem, and roots are typically ignored. At the physiological level though, a shoot-to-root-to-shoot loop has been described to drive sugar and cytokinin fluxes at floral transition in Arabidopsis relative white mustard^10^. In Arabidopsis, tagging of the FT protein with GFP demonstrated movement of the fusion protein from overexpressor scion into *ft* mutant rootstock^11^. In other species, FT-like proteins exported from the leaves can induce belowground processes such as tuberization in potato or bulb formation in onion^12^,^13^. All together these reports indicate that developmental signals originating from the leaf can reach the underground organs.

The roots may not only be involved in flowering by being on the route of systemic signals, but they may also participate actively in its regulation. To date, no dedicated study has been undertaken to examine this question: although numerous flowering time genes were identified, they were extensively studied in the shoot but not in the roots. In only a few cases did the analysis of expression patterns or phenotyping of Arabidopsis mutants include careful examination of the roots, followed subsequently by complementation tests^14^. This approach was used for *FT*, which is not expressed in the roots but whose partner *FD* is^15^. However, the root-specific expression of *FT* did not rescue the phenotype of the *ft* single mutant, indicating that the expression of *FT* in root tissues is not sufficient - albeit it might contribute - to flowering^15^. Other flowering time mutants show root architecture phenotypes^16^,^17^ and a major flowering QTL in Arabidopsis was associated with root xylem secondary growth^18^. However, whether those traits indicate root-specific functions or indirect effects of flowering time genes remains to be demonstrated.

To better understand the role of roots in the flowering process, we used two complementary approaches. First, we performed data mining of public microarray databases to obtain a global view of flowering-time genes expressed in the roots. Second, we analyzed the transcriptome of the roots during the induction of flowering. The set of differentially expressed genes was compared with publicly available datasets obtained in different contexts for uncovering potential regulatory networks.

## Results

### A majority of flowering-time genes are expressed in roots

**Figure 1:**
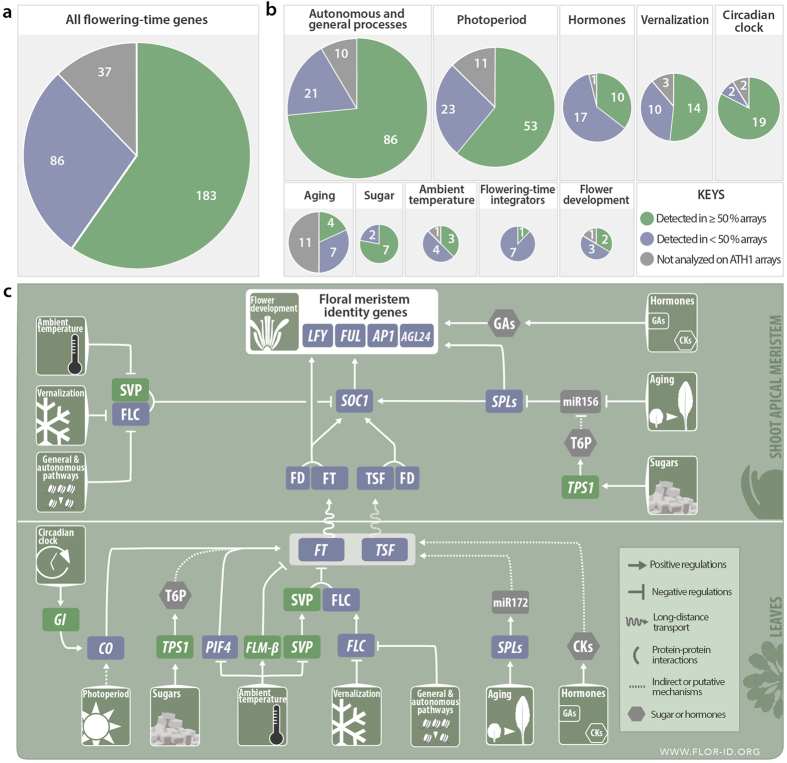
Flowering-time genes expressed in the roots *of Arabidopsis thaliana*. Genes expressed in the roots were identified by a present/absent call on 1,601 root ATH1 arrays retrieved from ArrayExpress repository (https://www.ebi.ac.uk/arrayexpress/). Flowering-time genes were extracted from FLOR-ID. **(a)** All 306 flowering genes. **(b)** Pie charts showing the same set of genes classified into flowering time pathways, circadian clock and flower development. Some genes are involved in more than one pathway. Pie chart area is proportional to gene number. **(c)** The snapshot of flowering pathways was extracted and adapted from FLOR-ID. Genes highlighted in green boxes were detected in ≥50% of root arrays. Genes in blue boxes were detected in <50 % arrays. Genes and compounds not analyzed in ATH1 arrays are in grey.

Data mining was performed using transcriptomic analyses of roots that are available in the ArrayExpress repository^19^ (Fig. 1). The set of selected experiments contained 1,601 Arabidopsis ATH1 Genome arrays (Supplementary Table 1). For each array, we performed an Affymetrix present/absent call and retained genes that were expressed (p < 0.01) in at least 50% of the 1,601 arrays. We used this criterion to filter out background or false positive genes, not ignoring that genes below the percentage cutoff likely include genes expressed in the roots but at low level or in specific experimental conditions. The filtered dataset contained 12,035 genes that we hereafter call “expressed in the roots”. We compared this dataset with a comprehensive list of 306 flowering-time genes that we established in the database FLOR-ID^1^. The flowering-time genes are allocated among different pathways whereby flowering occurs in response to photoperiod, vernalization, aging, ambient temperature, hormones, or sugar. An “autonomous pathway” leads to flowering independently of these signals and involves regulators of general processes such as chromatin remodeling, transcriptional machinery or proteasome activity. Eight genes under the control of several converging pathways are defined as “flowering-time integrators”: *FT*, *TSF*, *SUPPRESSOR OF OVEREXPRESSION OF CO1* (*SOC1*), *AGAMOUS*-*LIKE 24* (*AGL24*), *FRUITFULL* (*FUL*), *FLOWERING LOCUS C* (*FLC*), *SHORT VEGETATIVE PHASE* (*SVP*) and *LEAFY* (*LFY*). Given the design of ATH1 microarrays, 37 flowering-time genes including 11 genes encoding microRNAs could not be included in our survey because they are not represented in the probe set. Out of the 269 represented flowering time genes, 183 (68%) were expressed in roots in more than half of the analyzed arrays (Fig. 1a; Supplementary Table 2). Some flowering pathways were more enriched than others (Fig. 1b), *e.g.* the photoperiodic pathway, with 70% of its genes being expressed in the roots, and the sugar pathway with 7 genes out of 9 being active in the roots, including *TPS1*. Genes controlling autonomous flowering were widely detected in roots (80%). A side category of circadian clock genes was also highlighted in the analysis. By contrast, a low proportion of genes from the hormones and aging pathways were expressed.

By analyzing the data one at a time and focusing on the main flowering-time regulators highlighted in the overview snapshot of the FLOR-ID database^1^, we found that most of them were actually not expressed in the roots or at least did not pass the filter setting of being detected in at least 50% of the available root transcriptomes (Fig. 1c). In the photoperiod pathway, *CO* and *FT* were not detected in the dataset; only *GIGANTEA* (*GI*), which mediates the interaction between the clock and *CO* regulation^20^, was detected. The FT interactor FD and its paralogue FDP were only detected in 5% of the arrays. In the aging pathway, *MIRNA* genes were not analyzed on ATH1 arrays and their targets involved in flowering (the *SQUAMOSA*-*PROMOTER BINDING PROTEIN*-*LIKE*, *SPL*s) were not found in the majority of root microarrays. In the vernalization pathway, *FLC* was only detected in 11% of the arrays. As could be expected, flower meristem identity genes *LFY* and *APETALA1* (*AP1*) were not detected, but the upstream MADS box gene *SOC1* was expressed in 42% of the array.

The only pathways whose key regulators are clearly expressed in the roots are the sugar pathway, as *TPS1* was detected in 81% of the arrays, and the ambient temperature pathway, with *SVP* and *FLOWERING LOCUS M* (*FLM*) coming up in 73% and 51% of the arrays analyzed, respectively. This finding makes sense since all plant parts undoubtedly sense sugars and surrounding temperature, including the roots.

We can conclude from this global analysis that the flowering-time regulatory network involves a majority of genes, here estimated to more than two-thirds, expressed in the roots. The structure of the network in that part of the plant is however unpredictable since the expression of flowering-time integrators is not detected in most root-transcriptome analyses. Further experiments are thus required to link our analysis with functional data.

### Root transcriptome changes during the induction of flowering

**Figure 2:**
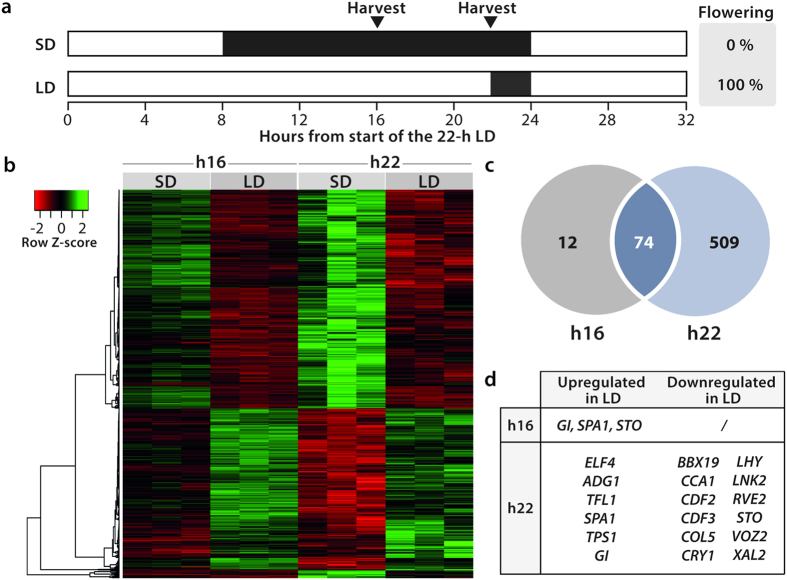
Root transcriptome changes during the induction of flowering by a single 22-h LD. **(a)** Experimental design. The proportion of plants having initiated flower buds two weeks after the experiment are shown on the right. **(b)** Heatmap of the differentially expressed genes (adjusted p-value ≤0.01; fold-change ≥2) showing three independent biological replicates per condition. Low expression levels in red, high expression levels in green. Relative expression values are scaled per transcript (lines). **(c)** Venn diagram of differentially expressed genes at both sampling time points. **(d)** List of differentially expressed flowering-time genes.

To identify new candidate genes expressed in the roots and potentially involved in flowering, we analyzed the root transcriptome during the induction of flowering (Fig. 2). Plants were grown in hydroponics for 7 weeks under 8-hour short days (8-h SD) and induced to flower by a single 22-hour long day (22-h LD)^21^. We harvested roots 16 and 22 h after the beginning of the 22-h LD and at the same times in control 8-h SD. Two weeks after the experiment, we dissected the remaining intact plants to check that those exposed to the 22-h LD had entered floral transition but not the 8-h SD controls (Fig. 2a). Three independent experiments were performed and used for a transcriptome analysis with Arabidopsis ATH1 arrays; the raw results were included in the data mining reported above. A total of 10,508 AGI loci passed filtering criteria and were considered to be expressed in the roots in our experimental system. This number is fully consistent with the size of the root-expressed gene dataset used in our data-mining analysis (12,035). The 10,508 genes included 168 flowering-time genes, among which 152 were common with the subset revealed by the global data mining shown in Fig. 1. Sixteen additional flowering-time genes were expressed in our experimental set-up, and therefore may be regulated by plant age or growing conditions (Supplementary Table 3). Among them, we found the floral integrator *SOC1* and two flowering-time genes involved in the control of meristem determinacy: *TERMINAL FLOWER 1* (*TFL1*), a gene in the same family as *FT* but which acts as a floral repressor in the shoot apical meristem^22^, and *XAANTAL2* (*XAL2*, also named *AGL14*), a gene involved in shoot and root development^23^,^24^.

The root transcriptome was found to undergo numerous changes during the inductive LD. At h16 (*i.e*. 8 hours from the extension of the photoperiod), 86 differentially expressed genes were identified in the roots and at h22, the number had increased to 583 (Fig. 2c). The heatmap shows that most changes occurring at h16 were actually amplified at h22 (74 of the 86 differentially expressed genes) (Fig. 2b) indicating that the experimental design targeted early events. In total, 595 differentially expressed genes were identified in the roots (Supplementary Table 3) among which 18 flowering time genes belonging to the photoperiod pathway, the circadian clock and the sugar pathway (Fig. 2d). This number represented about 10% of all the flowering time genes detected in the roots by data mining.

Members of the photoperiodic pathway included negative regulators of CO: *CYCLING DOF FACTOR2* and *3* (*CDF2*/*3*), *B-BOX DOMAIN PROTEIN 19* (*BBX19*) and *SUPPRESSOR OF PHYA-105 1* (*SPA1*) but whereas *CDF2/3* and *BBX19* were down-regulated in LD, *SPA1* was upregulated. Two positive regulators of CO were also up-regulated: *GI* and the blue-light photoreceptor gene *CRYPTOCHROME1* (*CRY1*). Two *CO*-like genes - *CONSTANS-LIKE5* (*COL5*) and *SALT TOLERANCE* (*STO*) - were down-regulated at h22 in LD, as well as the gene encoding the phytochrome B-interacting protein *VASCULAR PLANT ONE ZINC FINGER PROTEIN 2* (*VOZ2*).

Among clock components, several morning genes - *CIRCADIAN CLOCK ASSOCIATED1* (*CCA1*), *LATE ELONGATED HYPOCOTYL* (*LHY*), *NIGHT LIGHT-INDUCIBLE AND CLOCK-REGULATED2* (*LNK2*), and *REVEILLE2* (*RVE2*) - were repressed at h22 in LD. Conversely, two evening genes were upregulated: *GI* and *EARLY FLOWERING 4* (*ELF4*).

The prolonged photoperiod also induced the expression of two sugar metabolism-related genes: *TPS1* and *ADP GLUCOSE PYROPHOSPHORYLASE1* (*ADG1*), which encodes a subunit of ADP-glucose pyrophosphorylase (AGPase). Finally, we found that the expression of two genes involved in the control of meristem fate was also altered: *TFL1* was upregulated in LD whereas *XAL2* was repressed at h22.

### Differentially expressed genes are enriched in some root cell types and sugar signaling

**Figure 3:**
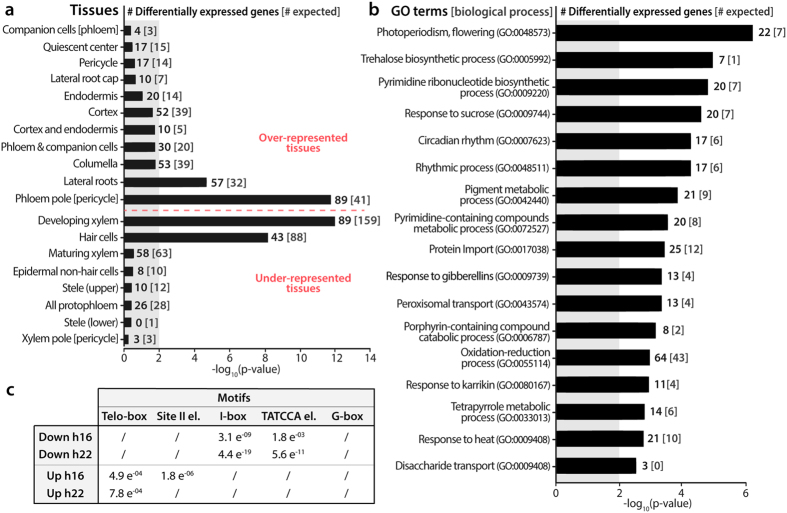
Enrichment analyses of the 595 genes differentially expressed in the roots during an inductive 22-h LD. (**a)** Tissue enrichment. For each gene, expression was localized in the tissue where Brady *et al*.^25^ found highest transcript level. In each tissue, the number of differentially expressed genes is indicated in bold whereas the number of genes that would be expected for this dataset is enclosed within brackets. Shaded area shows p-values >0.01. Over- and under-represented genes are separated by the horizontal dashed red line. **(b)** Gene ontology term enrichment in the list of 595 differentially expressed genes. GO identifiers are enclosed in brackets. The number of differentially expressed genes experimentally associated with each term is indicated in bold, whereas the number of genes associated with the GO term that would be expected by chance for this dataset is enclosed within brackets. Bars indicate the −log_10_(p-value) for each term (Fisher’s exact test). **(c)** Motif enrichment analysis in the −500 to +50 nt region of the genes that were down- or up-regulated at h16 or at h22 in LD. Numbers are the p-values of motifs that were identified as enriched by AME at p < 0.05 in any of the 4 differentially expressed gene subsets. / indicates non-enriched motif.

The list of 595 differentially expressed genes was thereafter submitted to different tests to see whether particular networks emerged. We performed three different searches based on (i) tissue enrichment, (ii) gene ontology and (iii) promoter sequences (Fig. 3).

First, we compared the list with the tissue-specific root transcriptome dataset published by Brady *et al*.^25^. As a reference, we used the whole set of 10,508 genes expressed in the roots in our experimental system, and found that they were mainly enriched in developing xylem and hair cells. This distribution was notably modified in the subset of differentially expressed genes with phloem pole-associated pericycle and lateral root initials being the cell populations where a significant part of the changes occurred (Fig. 3a).

Second, we performed a gene ontology enrichment test and found that ‘Photoperiodism, flowering’ was the most significantly enriched term in differentially expressed genes (Fig. 3b), followed by ‘Trehalose biosynthetic process’, ‘Pyrimidine ribonucleotide biosynthesis’, ‘Response to sucrose’, and ‘Circadian rhythm’.

Third, we searched for enriched cis-elements in the promoters of differentially expressed genes by using the MEME suite software (Fig. 3c). Differentially expressed genes were distributed among four subsets corresponding to their expression patterns (Fig. 2b): up or down in LD, at h16 or h22. A *de novo* motif search was performed with MEME (motif length between 8 and 15 nucleotides) and DREME (motif length ≤8) to find the most represented motifs in the promoters of each of the four gene subsets. Based on the study of Korkuc *et al*.^26^, we scanned the regions spanning −500 to +50 nt from the transcription start site of the genes. We found several close matches to five known cis-elements: the telo-box (AAACCC[TA]), the site II element (A[AG]GCCCA), the I-Box, the TATCCA element, and the G-box (CACGTG). To determine which of these motifs were specifically associated with the four expression patterns, we tested for the enrichment of each motif in the four subsets of differentially expressed genes with the AME tool. We found that both telo-box and site II elements were significantly enriched in upregulated genes and that I-Box and TATCCA were associated with repressed differentially expressed genes. The G-box was not significantly enriched in any subset (Fig. 3c).

### The change in photoperiod affects the root circadian clock

**Figure 4:**
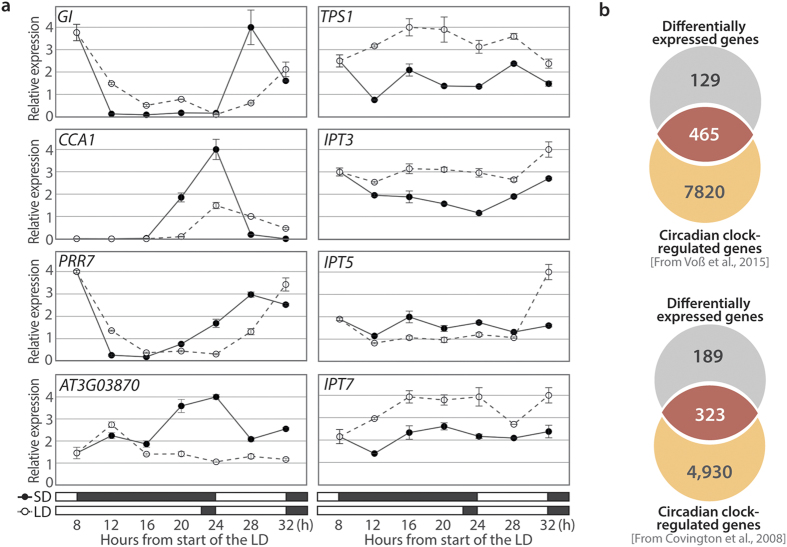
Temporal aspects of transcriptomic changes. **(a)** Time-course analyses of candidate gene expression. Relative transcript levels were analyzed by RT-qPCR during an 8-h SD (closed symbols) or a single 22-h LD (open symbols). Boxes in the bottom show light (white) and dark (black) periods. Data were normalized using *ACT2* and *UBQ10* genes. Error bars indicate the standard error of the mean for three experimental replicates. Data are from one representative experiment. **(b)** Estimate of circadian clock-regulated differentially expressed genes. Venn diagrams showing the overlap between the differentially expressed genes identified in this study and the circadian clock-regulated genes expressed in lateral roots [Dataset from Voß *et al*.^28^] or in the shoot [Dataset from Covington *et al*.^29^].

RT-qPCR analyses were performed on selected differentially expressed genes in order to confirm their differential expression (Fig. 4). Since several clock genes appeared on the list, we performed time-course experiments to verify the microarray results and evaluate to what extent circadian-regulated processes were affected by the photoperiodic treatment in greater detail. Roots were harvested every 4 h during the inductive 22-h LD and in control 8-h SD.

We analyzed the expression of *GI*, *CCA1* and *PSEUDO-RESPONSE REGULATOR 7* (*PRR7*) as representative clock genes^27^. The 22-h LD seemed to cause a 4-h delay in the expression patterns of these three genes, suggesting a phase shift of the circadian clock (Fig. 4a, left panel). Since such an effect could globally impact clock outputs, we attempted to evaluate the proportion of clock-regulated genes among the 595 differentially expressed genes. We therefore compared the list with datasets from transcriptomic analyses of circadian clock-regulated genes in lateral roots^28^ and shoot^29^. A large overlap of 78% and 63% was found with these datasets, respectively, revealing that the majority of the differentially expressed genes were indeed regulated by the circadian clock (Fig. 4b).

Our analysis also included an unknown gene (AT3G03870) that was downregulated at h16 and h22 in the microarray experiment as well as four candidate genes involved in sugar sensing and cytokinin biosynthesis (Fig. 4a, right panel). Most interestingly, *TPS1* was up-regulated in the roots during the 22-h LD. Our analysis also showed upregulation in LD of two *ISOPENTENYLTRANSFERASE* encoding genes (*IPT3* and *IPT7*) whereas a third one (*IPT5*) did not vary. These results confirmed the microarray data and suggested that sugar signaling and cytokinin biosynthesis were stimulated in the roots in response to the photoperiodic treatment.

### Reverse genetic analysis of differentially expressed genes did not reveal strong phenotypes

**Figure 5:**
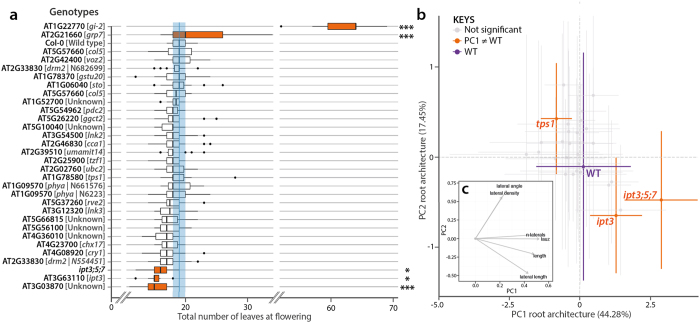
Flowering-time and root architecture phenotypes of selected mutants in 16-h LD. **(a)** Total number of leaves below the first flower (n = 15). * indicates a significant difference with WT Col-0 (Tukey’s HSD test, p < 0.05). *** indicates a highly significant difference with WT (Tukey’s HSD test, p < 0.01). WT is shown in blue. **(b)** Plot of the first two components of the Principal Component Analysis performed on root system architecture features. **(c)** Biplot of the two first components of the PCA. Orange color indicates significant differences with the WT Col-0. Supplementary Dataset 1 containing root phenotyping data is available at the following address: https://zenodo.org/record/50831.

We selected a subset of 30 differentially expressed genes for functional analyses, following a number of criteria, such as their expression fold change in the microarray analysis, their root-specific expression pattern (inferred from Covington *et al*. dataset^29^), their putative function, or their novelty (Supplementary Table 4). The corresponding available mutants were characterized for flowering-time and root architecture (Fig. 5). Only 5 mutants showed an altered flowering time phenotype in LD (Fig. 5a). Some of these mutants had been previously characterized, such as *gi-2,* which was very late flowering^30^, and *glycine-rich RNA-binding protein 7* (*grp7*, also called *ccr2*) which was only slightly delayed^31^. The cytokinin biosynthesis mutants *ipt3* and *ipt3;5;7* showed an early flowering phenotype, but the latter was highly pleiotropic^32^. Finally, the mutant for the *AT3G03870* gene of unknown function showed a weak early-flowering phenotype, producing 4 fewer leaves but bolting at the same time than Col-0 WT (Supplementary Table 5).

Since our selection of mutants resulted from transcriptomic analyses of roots, we closely examined the root phenotype of these mutants (Supplementary Dataset 1). In order to identify the genotypes whose root system significantly differed from WT, we performed a Principal Component Analysis (PCA) using different root architecture traits. The first two Principal Components (PC1 and PC2) were compared using Student tests with a threshold at p < 0.01. The mutants were then compared to WT for each variable (t-test, p < 0.01) (Fig. 5b). The PC1, which explained about 45 % of the variability of the dataset, reflects mostly the length of the primary root, the number of lateral roots, the length of the lateral roots, as well as the length of the apical unbranched zone of the primary root (Fig. 5c). The PC2 mainly reveals lateral root-related changes, such as their length, their insertion angle on the primary root as well as their density. Three mutants were statistically different from WT. The *tps1* mutant was affected in PC1 only, showing reduced length of the apical unbranched zone as well as shorter primary and lateral roots. The pleiotropic *ipt3;5;7* triple mutant showed a statistically different PC1, displaying an increased number and density of lateral roots. The *ipt3* single mutant also displayed a different PC1, albeit with a weaker lateral-root phenotype.

## Discussion

Molecular data concerning the involvement of the roots in the flowering process are lacking. Here, transcriptome analyses showed that at least 200 genes whose mutation had been shown to alter flowering time are expressed in the roots: 183 genes were identified in public resources and 16 additional genes popped-up in our experiments aiming to analyze the root transcriptome at the time of floral transition. This data-crossing relies on a hand-curated database of flowering-time genes^1^.

The small discrepancy in flowering-time gene numbers found in the two analyses is informative by the fact that some of these genes might be developmentally regulated in the roots. Indeed, most arrays deposited in databases were obtained from few-day old seedlings whereas we studied mature 7-week old plants. Among the 16 genes expressed in our hydroponics experiments but not reaching the 50% threshold in the data mining survey, we found genes regulating meristem determinacy in the shoot: *XAL2* and *TFL1*. Most interestingly, XAL2 is a direct regulator of *TFL1* expression in the shoot apical meristem but the genes have opposite effects on flowering time^22^,^24^. Both genes also have opposite effects on root growth: *XAL2* is necessary for normal patterning of root meristem, at least partly through auxin transport^23^, whereas *TFL1* was recently identified as a repressor of root growth^17^. We observed that the two genes were differentially expressed in the roots during the 22-h LD, but again in opposite ways: *XAL2* was down-regulated and *TFL1* was up-regulated, a situation that would delay flowering in the shoot and repress root growth. The upregulation of *TFL1* in the root is intriguingly similar to what is observed in the shoot meristem, where activation of *TFL1* at floral transition is important to counterbalance incoming flowering signals^33^. Whether this is relevant in the root requires further investigation.

In both the global and experimental microarray analyses, the photoperiodic pathway was found to be enriched in the roots and several regulators of *CO* were differentially expressed during the induction of flowering by one LD. Among them we found *CDF*s and *SPA1*, involved in the proteolysis of the CO protein. These results are striking since *CO* itself was not detected in the roots, confirming the very low level reported in other microarray studies^34^. In Takada and Goto^35^, some CO::*GUS* reporter lines showed an expression in the roots while others grown side by side did not, and hence uncertainty remains about *CO* pattern. Regulators of *CO* might have other putative targets in the roots, which remain to be discovered. Interestingly, two *CO*-like genes (*COL5* and *STO*) were found to be downregulated during the inductive LD but whether they share regulatory mechanisms with *CO* is currently unknown.

Some genes of the photoperiodic pathway that are expressed in the roots encode photoreceptors, such as *PHYTOCHROME A-B-C*, and *CRYPTOCHROME1-2* (Supplementary Table 2). Direct light effects on root growth are well documented and several reports therefore recommend to conduct experiments with roots in darkness^36^. However, a number of studies on root architecture in Arabidopsis are performed in transparent Petri dishes with all parts being illuminated. The majority of the root microarrays used for the data mining were obtained in these conditions (968 out of 1,601 arrays, Supplementary Table 1). We can then speculate that root illumination introduced a bias in the assembled dataset. By contrast, in our hydroponic device, roots were in complete darkness, hence we can assume that any light effect would be indirect. We tested this hypothesis by crossing our dataset with a transcriptomic analysis of seedling roots grown in the dark and exposed to 1-h red light^37^. After aligning the filter settings of Molas *et al*.’s analysis with ours, we found a very small overlap in gene expression: only 55 genes that were differentially expressed after the 1-h red light treatment^37^ were detected in the roots in our hydroponics device. This discrepancy further demonstrates that gene expression in roots is not directly impacted by light in our experiments. Therefore, we believe that even if some of the genes that are differentially expressed in the roots when the photoperiod is extended are known to be induced by light or to interact with different components of light signaling, this is not the primary reason for their differential expression. For example, *STO* and *ELF4*, two differentially-expressed flowering-time genes induced by light (Supplementary Table 6), also exhibit circadian expression patterns^38^,^39^,^40^. This is more likely the reason why they were differentially expressed in LD.

We estimated that around 70% of the genes that are differentially expressed in the roots during the 22-h LD are regulated by the circadian clock. This proportion is probably overestimated since it was calculated by comparing our dataset with public databases filtered with low stringency tools (see Materials and Methods). However, if one considers that approximately one-third of Arabidopsis transcripts are circadian-regulated^29^, the enrichment seems significant. Conceivably, differential expression of circadian-clock regulated genes might reflect changes in the period, the amplitude or the phase of the rhythms, which can respond to various inputs. Environmental factors are important since alterations in the environment due to the rotation of the earth has driven the evolution of the circadian clock, but a number of recent studies have demonstrated that the circadian clock is integrated very closely with primary metabolism^41^ as discussed below. Interestingly, developmental signals also come into play since the circadian clock was recently found to rephase during lateral root development^28^. In this context, it is worth highlighting that when we compared the set of differentially expressed genes in our experimental system and the cell-type transcriptomic data of Brady *et al*.^25^, we found enrichment in genes expressed in the lateral root initials (Fig. 3). This suggests that photoperiod and/or floral transition might affect root branching. We also found many changes in the transcriptome of phloem pole-associated pericycle cells, whereas lateral roots are derived from xylem-pole pericycle founder cells in Arabidopsis. These two populations of pericycle cells are known to display specific expression patterns and to be intimately associated with their underlying vascular tissue^42^. It is thus tempting to speculate that the changes in gene expression occurring in the phloem pole-associated pericycle cells during the extension of photoperiod were induced by signals coming from adjacent phloem. However, the marker used to sort phloem pole-associated pericycle cells, although capable of discriminating between the two populations of pericycle cells, was recently reported to be more widely expressed than initially thought^43^ and hence further verification is required.

To validate that circadian-regulated genes were especially affected by the photoperiodic change, we analyzed the expression of clock core genes in greater detail. The clock mechanism in Arabidopsis was shown to rely on three interlocking feedback loops^27^. The morning-phased loop comprises *PPR7* and *PPR9* and is activated by CCA1 and LHY; the evening-loop includes EARLY FLOWERING 3 (ELF3), ELF4 and LUX ARRYTHMO, which act together in an evening complex, and other evening genes including *GI* and *TIMING OF CAB EXPRESSION 1* (*TOC1*). The central loop makes the link between the morning and the evening loop since TOC1 activates *CCA1* and *LHY* whereas CCA1 and LHY proteins repress *TOC1*^27^.

Interestingly, we found that members of the evening loop - *GI* and *ELF4* - were upregulated whereas morning genes such as *CCA1* and *LHY* were downregulated in 22-h LD as compared to 8-h SD. These differential expression levels were recorded at two time points (h16 and h22) and were probably due to a delay in the expression patterns of these circadian genes upon extension of the photoperiod, as indicated by the time-course analyses (Fig. 4) and also reported in other studies^44^. Such changes might reflect the fact that the circadian clock in plants is entrained to light:dark cycles by photosynthetic inputs. It is known that sugars derived from photosynthesis entrain the circadian clock through morning genes in the shoot^45^ and that a shoot-derived photosynthesis product is necessary for the oscillation of the evening genes in the roots kept in darkness^46^. Moreover, the circadian clock orchestrates the coordinate adjustment of carbon partitioning and growth rate that occurs in response to photoperiod^47^. Consistently, we observed the differential expression of *ADG1*, encoding a subunit of AGPase involved in starch synthesis, and of *TPS1*, which catalyses formation of T6P, during the 22-h LD. T6P was found to mediate the sugar-dependent post-translational activation of AGPase^48^ and hence upregulation of *ADG1* and of *TPS1* might cooperatively stimulate starch synthesis in the roots during the extension of the photoperiod. Moreover, T6P was found to be positively correlated with rosette growth rate^49^ and to be required in the leaves and the shoot apical meristem at the time of flowering^4^. Altogether, our results suggest that roots are integrated in systemic signaling whereby carbon supply coordinates growth at a systemic level during the induction of flowering. This coordination possibly involves sugar input to the circadian clock and T6P pathway.

The involvement of sugar signaling is further supported by our *de novo* analysis of the promoters of genes upregulated at h16 and h22 during the 22-h LD. Both time points revealed an enrichment of the telo-box motif, which is present in the promoter of genes expressed in dividing cells of root meristems and is known to mediate the upregulation of glucose-responsive genes^50^. The telo-box, which would be part of a midnight regulatory module^51^, is frequently found to be associated with other motifs, such as the site II element^52^ that we also found in our analysis. The functional relevance of the association between these elements has been demonstrated for the SKIP-mediated control of root elongation^53^. Conversely, the promoters of genes downregulated during the 22-h LD were found to be enriched in both I-boxes, which are known to be part of a light regulatory module^54^, and in the sugar- and gibberellin-responsive element TATCCA, which is bound by MYB factors^55^. The TATCCA element and G-box were also found to be core components of the sugar response sequence (SRS) in the promoter of a sugar starvation–inducible rice α-amylase gene (Amy3)^56^. These results support a prominent role for sugars in the control of gene expression during the 22-h LD. Interestingly, we found that *IPT3* and *IPT7*, two cytokinin-biosynthesis genes expressed in the root vasculature and the endodermis^57^, were differentially expressed during the 22-h LD, whereas *IPT5*, which is expressed in the root cap, was not. An increased transport of cytokinins from the roots to the aerial part of the plant would establish a feedforward loop promoting flowering since these hormones are known to activate promoters of flowering in the shoot, such as *TSF* in the leaves and *SOC1* in the shoot apical meristem^9^. These mechanisms provide a molecular basis to the physiological shoot-to-root-to-shoot loop disclosed in the mustard *Sinapis alba* where sucrose arriving from the shoot induces cytokinin export from the roots to stimulate floral transition^10^.

In summary, our study sheds new insight into the involvement of the roots in the flowering process. Not only are a majority of flowering time genes expressed in the roots, but the root transcriptome displays important variations when plants are exposed to a photoperiodic treatment inducing flowering. These changes might be due to shoot signals, such as sugar, synchronizing root functioning with floral transition. At this stage, causal relationships can not be established since we know very little about the function of flowering time genes in the roots and what happens in the roots at the time of flowering. This study highlights the relevance of exploring the role of the roots in the flowering process.

## Materials and Methods

### Plant growth

Experiments were performed with *Arabidopsis thaliana* Col-0 accession. The *ipt3* and *ipt3;5;7* mutants were provided by Prof. Tatsuo Kakimoto (Osaka University, Japan); the *gi* mutant was given by Prof. George Coupland (Max Planck Institute for Plant Breeding Research, Köln, Germany). Other mutants were obtained from the Nottingham Arabidopsis Stock Center (http://www.arabidopsis.info). Accession numbers are provided in Supplementary Table 4. All seeds were bulked at the same time to reduce variability. Plants were grown in a hydroponic device made of black containers and accessories (http://www.araponics.com). Nutrient solution was a mix of commercial stocks (0.5 ml l^−1^ FloraMicro, FloraGro and FloraBloom; http://www.generalhydroponics.com). Light was provided by fluorescent white tubes at 60 μE.m^−2^.s^−1^ PPFD; temperature was 20 °C (day/night) and air relative humidity 70%. For transcriptomic analyses in WT plants, flowering was induced by a single 22-h LD after 7 weeks in 8-h SD and the flowering response was scored as the % of plants having initiated floral buds two weeks after the LD^21^. For mutant phenotyping, plants were cultivated in 16-h LD and the total number of leaves below the first flower (rosette + cauline leaves) was scored to estimate flowering time.

### Microarray analysis

The total root system of 7 week-old plants (n = 18) was harvested 16 h and 22 h after the beginning of the inductive LD and pooled. Sampling at the same times in 8-h SD happened during the dark period and was performed under dim green light. Roots were stored at −80 °C until used. Tissues were ground in liquid nitrogen and RNA was extracted with TRizol according to manufacturer’s instructions (www.lifetechnologies.com). We assessed RNA integrity with the Experion^tm^ automated electrophoresis system (www.bio-rad.com). All the samples used for microarray analysis had maximum RNA quality indicator (RQI) values of 10. The RNA samples were labeled using 3′ IVT Expressed kit according to the manufacturer’s instructions (Affymetrix, www.affymetrix.com). Three biological replicates obtained from independent experiments were hybridized on ATH1 Genome arrays (Affymetrix). We analyzed raw data using the limma package^58^. Data were GCRMA-normalized, probesets were filtered for an absolute expression level of at least 100 in ≥20% of the arrays, and data were fitted to a linear model using the lmfit() function. The statistics for differential expression was computed using the ebayes() function, and we corrected the p-value for multiple testing (false discovery rate adjustement using the Benjamini-Hochberg procedure)^59^. We considered genes as being differentially expressed when the adjusted p-value was ≤0.01 and fold-change ≥2.

### *In silico* analysis

#### Data mining

*In silico* transcriptomic analyses were performed on Arabidopsis Affymetrix ATH1 raw data retrieved from ArrayExpress (http://www.ebi.ac.uk/arrayexpress/) using the query “roots”. The resulting list was manually sorted to remove experiments lacking comprehensive methodological information. Each experiment was manually curated to select root-specific raw files. The list of experiments included in the survey is available in Supplementary Table 1. The subsequent analysis was performed using the R programming language. The “simpleaffy” Bioconductor package V.2.44.0^60^ was used to read the raw data and perform the present/absent call on individual arrays using the detection.p.val() function. Genes were considered as being expressed when p-value < 0.01. We computed the proportion of arrays in which expression of the gene of interest could be detected.

#### Experimental microarray analyses

The analysis of tissue enrichment was performed from dataset published in Brady *et* h gene represented in the ATH1 arrays was associated with the tissue where its expression was maximal in Brady’s study. The resulting map was used to localize the genes identified in our study and to calculate their distribution among the tissues of the roots. This exercise was performed with the list of all root-expressed genes (expression level of at least 100 in ≥20% of the arrays) or the genes differentially expressed during the photoperiodic induction of flowering (adjusted p-value ≤0.01). Using the resulting data, we performed a Fisher’s exact test to determine whether tissues were over- or under-represented in the differentially expressed genes list; the tissues in which the number of differentially expressed genes was higher than expected were tested for over-representation while tissues in which the number of differentially expressed genes was lower than expected were tested for under-representation (p-value ≤0.01).

The Gene Ontology Enrichment analysis was performed using the topGO package V2.20.0 with the annotation of the ATH1 array from ath1121501.db package V3.1.4. We performed a Biological Process (BP) enrichment analysis using the classic Fisher’s exact test (p < 0.001). Redundant GO terms were removed using REVIGO^61^ (http://revigo.irb.hr). The expected numbers of differentially expressed genes were computed based both on the total number of root-expressed genes (see above) and the number of differentially expressed genes in our microarray analysis.

The analysis of clock-regulated genes exploited datasets obtained in studies of the circadian clock in shoots^29^ and lateral roots^28^. Covington and colleagues analyzed different publically available circadian microarray datasets; we used their largest list. In Voß’s study, the authors identified highly-probable circadian clock-regulated genes in the roots using three different analysis tools. We used the list of genes predicted to be clock-regulated by at least one of those tools. When we crossed our experimental list of differentially expressed genes with these datasets, we found that some differentially expressed genes were not represented in Covington’s or Voß’s arrays and hence were excluded for the comparison.

### RT-qPCR analysis

The total root system of 7 week-old plants (n = 18) was harvested every 4 h during the 22-h LD and at the same times in control 8-h SD. Roots were stored at −80 °C until used. Tissues were ground in liquid nitrogen and RNA was extracted with TRizol according to manufacturer’s instructions (www.lifetechnologies.com). RNA samples were treated with DNase (0.2 U DNase μg^−1^). We synthesized first-strand cDNA from 1.5 μg RNA using MMLV reverse transcriptase and oligo(dT)_15_ according to manufacturer’s instructions (http://www.promega.com). Quantitative PCR (qPCR) reactions were performed in triplicates using SYBR-Green I (http://www.eurogentec.com) in 96-well plates with an iCycler IQ5 (http://www.bio-rad.com). We extracted quantification cycle (Cq) values using the instrument software and imported the data in qbase^PLUS^ 2.0 (http://www.biogazelle.com). A GeNorm analysis^62^ was performed in a preliminary experiment to identify suitable reference genes. We selected *ACTIN2* (*ACT2*) and *TUBULIN2* (*TUB2*) (geNorm M value <0.2). The computed geometric mean of their Cq values was used to calculate the normalization factor, as in Vandesompele *et al*.^62^. Primers are listed in Supplementary Table 7.

### Root phenotyping

Plants used for root architecture analysis were grown *in vitro* on 0.5 × MS, 1% sucrose; sterilized seeds were sown after three days of stratification at 4 °C. Square Petri dishes were used and placed vertically, under 100 μE.m^−2^.s^−1^ PPFD, in 16-h LD, at 20 °C. Root pictures were taken nine days after sowing using a CCD camera (Canon EOS 1100D with a Canon Lense EF 50 mm 1:1.8) and analyzed using the ImageJ plugin “SmartRoot”^63^. Root tracings were exported and analysed in R. For genotype comparison, we performed a Principal Component Analysis (PCA) using the length of the primary root, the length of the apical unbranched zone and lateral root features: number, density, total length and angle. The resulting PC’s were compared using Student tests with a threshold at p < 0.01. The selected genotypes were then compared to WT for each variable (t-test, p < 0.01). Data visualization was performed using ggplot2 package^64^.

### Cis-elements analysis

For each subset of similarly controlled genes, we prepared a fasta formated file containing the promoter sequences (−500, +50) obtained from the TAIR10 ftp repository^65^. The analyses were performed using the command line version of the MEME-Suite^66^ (http://meme-suite.org, version 4.10.0). The parameters for MEME were set as default values, except for: maximum width of each motif: 15 bp; maximum number of motifs to find: 10; background sequences: all TAIR10 promoters (−500, +50). The parameters for DREME and AME were set as default values, with the background sequences being the promoters of the 10,508 genes found to be expressed in the roots.

## Additional Information

**Accession codes:** Microarray data are available in the ArrayExpress database (http://www.ebi.uk/arrayexpress) with the accession numbers E-MTAB-4129 and E-MTAB-4130.

**How to cite this article**: Bouché, F. *et al*. Integrating roots into a whole plant network of flowering time genes in *Arabidopsis thaliana*. *Sci. Rep.* **6**, 29042; doi: 10.1038/srep29042 (2016).

## Supplementary Material

Supplementary Information

Supplementary Table S1

Supplementary Table S2

Supplementary Table S5
