# Supplementary material for: Integrating roots into a whole plant network of flowering time genes in Arabidopsis thaliana
Source: Sci Rep. 2016 Jun 29;6:29042. doi: 10.1038/srep29042 (PMC4926122; doi:10.1038/srep29042)
Supplement: Supplementary Information [file srep29042-s1.pdf]

Integrating roots into a whole plant network of flowering time genes in *Arabidopsis thaliana*

Frédéric Bouché [1,2], Maria D'Aloia [1,3], Pierre Tocquin [1],
Guillaume Lobet [1], Nathalie Detry [1], and Claire Périlleux [1*].

[1] PhytoSYSTEMS, Laboratory of Plant Physiology, University of Liège,
Quartier Vallée 1 Sart Tilman Campus, Chemin de la Vallée n°4, B-4000 Liège, Belgium.

[2] Current address: Department of Biochemistry, University of Wisconsin-Madison,
433 Babcock Drive, Madison, WI 53706-1544, USA

[3] Current address : GlaxoSmithKline Biologicals, Research & Development,
Avenue Fleming 20, 1300 Wavre, Belgium

* For correspondence (Tel: +32 4 3663833, e-mail cperilleux@ulg.ac.be)

Supplementary Table 1: List of root microarrays used for data mining

GEOD	Media	Age (d)	Photoperiod	Purpose	Only roots in the dataset ? If not, the non-root array were removed	GEOD	Number of arrays	Raw data
E-ATMX-31	Hydroponics	21	16	General root transcriptome.	NO	E-ATMX-31	9	http://www.ebi.ac.uk/arrayexpress/files/E-ATMX-31/E-ATMX-31.raw.1.zip
E-GEOD-15189	Hydroponics	30	10	Response to Fe deprivation.	YES (whole roots)	E-GEOD-15189	22	http://www.ebi.ac.uk/arrayexpress/files/E-GEOD-15189/E-GEOD-15189.raw.1.zip
E-GEOD-22114	Hydroponics	28	16	Response to cadmium (200µM) treatment.	YES (whole roots)	E-GEOD-22114	6	http://www.ebi.ac.uk/arrayexpress/files/E-GEOD-22114/E-GEOD-22114.raw.1.zip
E-GEOD-24348	Hydroponics	35	16	*nas4x* mutant profiling after iron deprivation.	NO	E-GEOD-24348	24	http://www.ebi.ac.uk/arrayexpress/files/E-GEOD-24348/E-GEOD-24348.raw.1.zip
E-GEOD-29086	Hydroponics	24	16	Profiling of *kas*-1 and *Tsu* -1 mutants after iron deprivation.	YES (whole roots)	E-GEOD-29086	18	http://www.ebi.ac.uk/arrayexpress/files/E-GEOD-29086/E-GEOD-29086.raw.1.zip
E-GEOD-29589	Hydroponics	20	16	Response to nitrate and ammonium.	YES (whole roots)	E-GEOD-29589	15	http://www.ebi.ac.uk/arrayexpress/files/E-GEOD-29589/E-GEOD-29589.raw.1.zip
E-GEOD-33790	Hydroponics	30	12	Response to phosphate starvation.	NO	E-GEOD-33790	18	http://www.ebi.ac.uk/arrayexpress/files/E-GEOD-33790/E-GEOD-33790.raw.1.zip
E-GEOD-34130	Hydroponics	12	ND	Response to nitrogen in different *A. thaliana* accessions.	YES (whole roots)	E-GEOD-34130	42	http://www.ebi.ac.uk/arrayexpress/files/E-GEOD-34130/E-GEOD-34130.raw.1.zip
E-GEOD-35544	Hydroponics	15	16	Root nitrate response of Ws plants and *afb3-1* mutant.	YES (whole roots)	E-GEOD-35544	12	http://www.ebi.ac.uk/arrayexpress/files/E-GEOD-35544/E-GEOD-35544.raw.1.zip
E-GEOD-36789	Hydroponics	30	12	Response to high osmotic stress.	NO	E-GEOD-36789	18	http://www.ebi.ac.uk/arrayexpress/files/E-GEOD-36789/E-GEOD-36789.raw.1.zip
E-GEOD-43011	Hydroponics	14	16	Response of *tga1/tga4* mutant to nitrate.	YES (whole roots)	E-GEOD-43011	12	http://www.ebi.ac.uk/arrayexpress/files/E-GEOD-43011/E-GEOD-43011.raw.1.zip
E-GEOD-46958	Hydroponics	ND	16	Response to gold treatment (0.125mM).	YES (whole roots)	E-GEOD-46958	6	http://www.ebi.ac.uk/arrayexpress/files/E-GEOD-46958/E-GEOD-46958.raw.1.zip
E-GEOD-6155	Hydroponics	28	24	Profiling of *ANR1* overexpressor.	YES (whole roots)	E-GEOD-6155	7	http://www.ebi.ac.uk/arrayexpress/files/E-GEOD-6155/E-GEOD-6155.raw.1.zip
E-GEOD-7631	Hydroponics	12	16	Tissue-speicific response to KNO3.	YES (different cell types)	E-GEOD-7631	84	http://www.ebi.ac.uk/arrayexpress/files/E-GEOD-7631/E-GEOD-7631.raw.1.zip
Present_work	Hydroponics	42	10	Root profilling.	YES (whole roots)	ND	3	http://www.ebi.ac.uk/arrayexpress/files/E-MTAB-4129
Present_work	Hydroponics	49	8	Response of root to photoperiodic induction of flowering.	YES (whole roots)	ND	14	http://www.ebi.ac.uk/arrayexpress/files/E-MTAB-4130
E-GEOD-18984	In_vitro_liquid_media	10	24	Response to nitrate.	NO	E-GEOD-18984	8	http://www.ebi.ac.uk/arrayexpress/files/E-GEOD-18984/E-GEOD-18984.raw.1.zip
E-GEOD-19978	In_vitro_liquid_media	7	24	Response to CLAVATA3/ESR-related peptides.	YES (Root tip)	E-GEOD-19978	6	http://www.ebi.ac.uk/arrayexpress/files/E-GEOD-19978/E-GEOD-19978.raw.1.zip
E-GEOD-51236	In_vitro_liquid_media	26	ND	Anaylsis of *mft1* mutant's roots.	YES (whole roots)	E-GEOD-51236	9	http://www.ebi.ac.uk/arrayexpress/files/E-GEOD-51236/E-GEOD-51236.raw.1.zip
E-MEXP-3649	In_vitro_liquid_media	17	16	Iron deficiency in *myb10;myb72* double KO.	YES (whole roots)	E-MEXP-3649	4	http://www.ebi.ac.uk/arrayexpress/files/E-MEXP-3649/E-MEXP-3649.raw.1.zip
E-GEOD-19245	In_vitro_then_hydroponics	28	16	Response to Cadmium chloride treatment.	NO	E-GEOD-19245	24	http://www.ebi.ac.uk/arrayexpress/files/E-GEOD-19245/E-GEOD-19245.raw.1.zip
E-GEOD-5620	In_vitro_then_liquid_media	17	16	Profiling of roots and shoots.	NO	E-GEOD-5620	36	http://www.ebi.ac.uk/arrayexpress/files/E-GEOD-5620/E-GEOD-5620.raw.1.zip
E-GEOD-5622	In_vitro_then_liquid_media	17	16	Response to osmotic treatment.	NO	E-GEOD-5622	24	http://www.ebi.ac.uk/arrayexpress/files/E-GEOD-5622/E-GEOD-5622.raw.1.zip
E-GEOD-5623	In_vitro_then_liquid_media	17	16	Response to salt stress.	NO	E-GEOD-5623	24	http://www.ebi.ac.uk/arrayexpress/files/E-GEOD-5623/E-GEOD-5623.raw.1.zip
E-GEOD-5624	In_vitro_then_liquid_media	17	16	Response to drought stress.	NO	E-GEOD-5624	28	http://www.ebi.ac.uk/arrayexpress/files/E-GEOD-5624/E-GEOD-5624.raw.1.zip
E-GEOD-5625	In_vitro_then_liquid_media	17	16	Response to bleomycin and mitomycin.	NO	E-GEOD-5625	24	http://www.ebi.ac.uk/arrayexpress/files/E-GEOD-5625/E-GEOD-5625.raw.1.zip
E-GEOD-5626	In_vitro_then_liquid_media	17	16	Response to UV-B radiations.	NO	E-GEOD-5626	28	http://www.ebi.ac.uk/arrayexpress/files/E-GEOD-5626/E-GEOD-5626.raw.1.zip
E-GEOD-5627	In_vitro_then_liquid_media	17	16	Response to wounding.	NO	E-GEOD-5631	21	http://www.ebi.ac.uk/arrayexpress/files/E-GEOD-5627/E-GEOD-5627.raw.1.zip
E-GEOD-5628	In_vitro_then_liquid_media	17	16	Response to heat.	NO	E-GEOD-5628	32	http://www.ebi.ac.uk/arrayexpress/files/E-GEOD-5628/E-GEOD-5628.raw.1.zip
E-MEXP-2601	Sand_pots	45	8	Response to phosphate.	YES (whole roots)	E-MEXP-2601	12	http://www.ebi.ac.uk/arrayexpress/files/E-MEXP-2601/E-MEXP-2601.raw.1.zip
E-MEXP-828	Sand_pots	42	8	Response to nitrate/sucrose.	YES (whole roots)	E-MEXP-828	34	http://www.ebi.ac.uk/arrayexpress/files/E-MEXP-828/E-MEXP-828.raw.1.zip
E-MTAB-1582	Sand_pots	56	ND	Competition with other species (*H. Pilosella*).	YES (whole roots)	E-MTAB-1582	6	http://www.ebi.ac.uk/arrayexpress/files/E-MTAB-1582/E-MTAB-1582.raw.1.zip
Present_work	Soil	42	10	Root profilling.	YES (whole roots)	ND	3	http://www.ebi.ac.uk/arrayexpress/files/E-MTAB-4129
E-GEOD-10496	Solid_MS	6	ND	Comparison of whole root and FACS sorted cell roots.	YES (Whole roots vs FACS)	E-GEOD-10496	6	http://www.ebi.ac.uk/arrayexpress/files/E-GEOD-10496/E-GEOD-10496.raw.1.zip
E-GEOD-10497	Solid_MS	5	ND	Response to iron deprivation.	YES (Root sections)	E-GEOD-10497	16	http://www.ebi.ac.uk/arrayexpress/files/E-GEOD-10497/E-GEOD-10497.raw.1.zip
E-GEOD-10501	Solid_MS	5	ND	Response to iron deprivation.	YES (different tissues)	E-GEOD-10501	16	http://www.ebi.ac.uk/arrayexpress/files/E-GEOD-10501/E-GEOD-10501.raw.1.zip
E-GEOD-10502	Solid_MS	5	ND	Response to iron deprivation.	YES (whole roots)	E-GEOD-10502	14	http://www.ebi.ac.uk/arrayexpress/files/E-GEOD-10502/E-GEOD-10502.raw.1.zip
E-GEOD-10576	Solid_MS	6	ND	Tissue-specific reponse to iron deprivation.	YES (different tissues)	E-GEOD-10576	52	http://www.ebi.ac.uk/arrayexpress/files/E-GEOD-10576/raw/
E-GEOD-11558	Solid_MS	14	12	Response to oxigen concentrations.	YES (whole roots)	E-GEOD-11558	28	http://www.ebi.ac.uk/arrayexpress/files/E-GEOD-11558/E-GEOD-11558.raw.1.zip
E-GEOD-14502	Solid_MS	7	16	Response to hypoxia.	NO	E-GEOD-14502	79	http://www.ebi.ac.uk/arrayexpress/files/E-GEOD-14502/E-GEOD-14502.raw.1.zip
E-GEOD-16468	Solid_MS	6	16	Profilling of root tissues.	YES (different cell types)	E-GEOD-16468	8	http://www.ebi.ac.uk/arrayexpress/files/E-GEOD-16468/E-GEOD-16468.raw.1.zip
E-GEOD-16469	Solid_MS	6	16	Endodermis profiling.	YES (only endodermis)	E-GEOD-16469	3	http://www.ebi.ac.uk/arrayexpress/files/E-GEOD-16469/E-GEOD-16469.raw.1.zip
E-GEOD-20223	Solid_MS	7	24	Response to Gibberellin.	YES (Root tip)	E-GEOD-20223	6	http://www.ebi.ac.uk/arrayexpress/files/E-GEOD-20223/E-GEOD-20223.raw.1.zip
E-GEOD-20226	Solid_MS	45	8	Response to Phytophthora parasitica.	YES (whole roots)	E-GEOD-20226	10	http://www.ebi.ac.uk/arrayexpress/files/E-GEOD-20226/E-GEOD-20226.raw.1.zip
E-GEOD-20232	Solid_MS	14	ND	*arr10* and *arr12* mutant response to cytokinin (trans-zeatin).	YES (whole roots)	E-GEOD-20232	12	http://www.ebi.ac.uk/arrayexpress/files/E-GEOD-20232/E-GEOD-20232.raw.1.zip
E-GEOD-20493	Solid_MS	18	16	Profiling of Fd-*gogat1*/*glu1* mutant.	NO	E-GEOD-20493	16	http://www.ebi.ac.uk/arrayexpress/files/E-GEOD-20493/E-GEOD-20493.raw.1.zip
E-GEOD-21443	Solid_MS	7	16	Response of pye mutant to iron deprivation.	YES (whole roots)	E-GEOD-21443	8	http://www.ebi.ac.uk/arrayexpress/files/E-GEOD-21443/E-GEOD-21443.raw.1.zip
E-GEOD-22966	Solid_MS	13	16	Responses to NO3- heterogenous environment.	YES (whole roots)	E-GEOD-22966	36	http://www.ebi.ac.uk/arrayexpress/files/E-GEOD-22966/E-GEOD-22966.raw.1.zip
E-GEOD-27475	Solid_MS	14	16	Knock-down of *erf3/hre1* under hypoxy.	YES (whole roots)	E-GEOD-27475	16	http://www.ebi.ac.uk/arrayexpress/files/E-GEOD-27475/E-GEOD-27475.raw.1.zip
E-GEOD-28275	Solid_MS	5	16	Analysis of introgression lines on tilted plates.	YES (Root tip)	E-GEOD-28275	9	http://www.ebi.ac.uk/arrayexpress/files/E-GEOD-28275/E-GEOD-28275.raw.1.zip
E-GEOD-30091	Solid_MS	5	ND	Response to low pH (4.6 vs 5.7).	YES (whole roots)	E-GEOD-30091	5	http://www.ebi.ac.uk/arrayexpress/files/E-GEOD-30091/E-GEOD-30091.raw.1.zip
E-GEOD-30095	Solid_MS	6	ND	Response to low pH (4.6 vs 5.7).	YES (different tissues)	E-GEOD-30095	30	http://www.ebi.ac.uk/arrayexpress/files/E-GEOD-30095/E-GEOD-30095.raw.1.zip
E-GEOD-30096	Solid_MS	6	ND	Response to low pH (4.6 vs 5.7).	YES (Root sections)	E-GEOD-30096	16	http://www.ebi.ac.uk/arrayexpress/files/E-GEOD-30096/E-GEOD-30096.raw.1.zip
E-GEOD-30097	Solid_MS	5	ND	Response to low pH (4.6 vs 5.7).	YES (whole roots)	E-GEOD-30097	28	http://www.ebi.ac.uk/arrayexpress/files/E-GEOD-30097/E-GEOD-30097.raw.1.zip
E-GEOD-30098	Solid_MS	5	ND	Response to sulphur deprivation.	YES (whole roots)	E-GEOD-30098	12	http://www.ebi.ac.uk/arrayexpress/files/E-GEOD-30098/E-GEOD-30098.raw.1.zip
E-GEOD-30099	Solid_MS	5	ND	Response to sulphur deprivation.	YES (different tissues)	E-GEOD-30099	15	http://www.ebi.ac.uk/arrayexpress/files/E-GEOD-30099/E-GEOD-30099.raw.1.zip
E-GEOD-30166	Solid_MS	6	ND	Response to low pH and sulfur deficiency.	YES (different tissues)	E-GEOD-30166	116	http://www.ebi.ac.uk/arrayexpress/files/E-GEOD-30166/raw/
E-GEOD-32659	Solid_MS	5	16	Response to Boron toxicity.	YES (whole roots)	E-GEOD-32659	6	http://www.ebi.ac.uk/arrayexpress/files/E-GEOD-32659/E-GEOD-32659.raw.1.zip
E-GEOD-35580	Solid_MS	7	18	Tissue-specific response to auxin (IAA 5 µM).	YES (different tissues)	E-GEOD-35580	30	http://www.ebi.ac.uk/arrayexpress/files/E-GEOD-35580/E-GEOD-35580.raw.1.zip
E-GEOD-42007	Solid_MS	6	24	Response to auxin (IAA 1 µM).	YES (whole roots)	E-GEOD-42007	48	http://www.ebi.ac.uk/arrayexpress/files/E-GEOD-42007/E-GEOD-42007.raw.1.zip
E-GEOD-42896	Solid_MS	3	24	Lateral root induction analysis on NAA and Naxillin.	YES (whole roots)	E-GEOD-42896	15	http://www.ebi.ac.uk/arrayexpress/files/E-GEOD-42896/E-GEOD-42896.raw.1.zip
E-GEOD-46205	Solid_MS	6	16	Response to NaCl (140 mM).	YES (different tissues)	E-GEOD-46205	96	http://www.ebi.ac.uk/arrayexpress/files/E-GEOD-46205/E-GEOD-46205.raw.1.zip
E-GEOD-52208	Solid_MS	12	16	Response to boron limitation (0.3 vs 100 µM).	YES (whole roots)	E-GEOD-52208	14	http://www.ebi.ac.uk/arrayexpress/files/E-GEOD-52208/E-GEOD-52208.raw.1.zip
E-GEOD-52610	Solid_MS	6	16	ATP effect on Arabidopsis roots in dorn1-1 mutant.	YES (whole roots)	E-GEOD-52610	12	http://www.ebi.ac.uk/arrayexpress/files/E-GEOD-52610/E-GEOD-52610.raw.1.zip
E-GEOD-7636	Solid_MS	6	ND	Comparison of whole root and FACS sorted cell roots.	YES (Whole roots vs FACS)	E-GEOD-7636	4	http://www.ebi.ac.uk/arrayexpress/files/E-GEOD-7636/E-GEOD-7636.raw.1.zip
E-GEOD-7639	Solid_MS	6	ND	Transversal sections response to NaCl treatment (140 mM).	YES (Root sections)	E-GEOD-7639	16	http://www.ebi.ac.uk/arrayexpress/files/E-GEOD-7639/E-GEOD-7639.raw.1.zip
E-GEOD-7641	Solid_MS	6	ND	Tissue-specific reponse to NaCl treatment (140 mM).	YES (different cell types)	E-GEOD-7641	36	http://www.ebi.ac.uk/arrayexpress/files/E-GEOD-7641/E-GEOD-7641.raw.1.zip
E-GEOD-8787	Solid_MS	6	ND	Response to NaCl (140 mM).	YES (Root tip)	E-GEOD-8787	16	http://www.ebi.ac.uk/arrayexpress/files/E-GEOD-8787/E-GEOD-8787.raw.1.zip
E-GEOD-9311	Solid_MS	10	16	Response to Selenium.	NO	E-GEOD-9311	8	http://www.ebi.ac.uk/arrayexpress/files/E-GEOD-9311/E-GEOD-9311.raw.1.zip
E-MEXP-2140	Solid_MS	5	16	Profiling of *pickle* mutants.	YES (Root tip)	E-MEXP-2140	12	http://www.ebi.ac.uk/arrayexpress/files/E-MEXP-2140/E-MEXP-2140.raw.1.zip
E-MEXP-2483	Solid_MS	11	16	Profiling of *fry1* mutants.	NO	E-MEXP-2483	8	http://www.ebi.ac.uk/arrayexpress/files/E-MEXP-2483/E-MEXP-2483.raw.1.zip
E-MEXP-2912	Solid_MS	7	24	Profiling og *arf7;arf19* double mutant.	YES (different tissues)	E-MEXP-2912	30	http://www.ebi.ac.uk/arrayexpress/files/E-MEXP-2912/E-MEXP-2912.raw.1.zip
E-MEXP-635	Solid_MS	6	24	Profiling of *brx* mutant and corresponding rescue line.	YES (whole roots)	E-MEXP-635	6	http://www.ebi.ac.uk/arrayexpress/files/E-MEXP-635/E-MEXP-635.raw.1.zip
E-MEXP-791	Solid_MS	10	14	Response to phosphate.	NO	E-MEXP-791	24	http://www.ebi.ac.uk/arrayexpress/files/E-MEXP-791/E-MEXP-791.raw.1.zip
E-GEOD-19242	Solid_MS	14	16	*cys-c1* mutant profiling.	YES (whole roots)	E-GEOD-19242	6	http://www.ebi.ac.uk/arrayexpress/files/E-GEOD-19242/E-GEOD-19242.raw.1.zip
E-GEOD-40076	Solid_MS	10	16	response to iron (100 µM).	NO	E-GEOD-40076	24	http://www.ebi.ac.uk/arrayexpress/files/E-GEOD-40076/E-GEOD-40076.raw.1.zip

Supplemental Table 4: List of mutants characterized in this study

AGI identifier	Gene name	SALK identifier	NASC identifier	Microarray results	
				Hour 16	Hour 22
**AT5G57660**	*COL5*	SALK_137717C	N661168	Not Diff. Expressed	DOWN
**AT4G08920**	*CRY1*	SALK_042397C	N662234	Not Diff. Expressed	DOWN
**AT2G46830**	*CCA1*	SALK_146072C	N677310	Not Diff. Expressed	DOWN
**AT5G37260**	*RVE2*	SALK_051842C	N685922	Not Diff. Expressed	DOWN
**AT2G42400**	*VOZ2*	SALK_115813C	N656888	Not Diff. Expressed	DOWN
**AT1G09570**	*PHYA*	SALK_014575C	N661576	Not Diff. Expressed	DOWN
**AT1G09570**	*PHYA* (*phya-211*)	/	N6223	Not Diff. Expressed	DOWN
**AT2G02760**	*UBC2*	SALK_152462C	N669285	Not Diff. Expressed	DOWN
**AT1G06040**	*STO*	SALK_067473C	N655591	UP	DOWN
**AT2G21660**	*GRP7*	SALK_039556C	N682254	Not Diff. Expressed	UP
**AT1G78580**	*TPS1*	SALK_142995C	N657845	Not Diff. Expressed	UP
**AT1G22770**	*GI* (*gi-2*)	Kindly provided by Prof. Coupland.		UP	UP
**AT1G78370**	*GSTU20*	SALK_091292C	N663302	Not Diff. Expressed	UP
**AT3G54500**	*LNK2*	SALK_141609C	N686901	Not Diff. Expressed	DOWN
**AT3G12320**	*LNK3*	SALK_085551C	N666599	Not Diff. Expressed	DOWN
**AT2G33830**	*DRM2*	SALK_098437C	N682699	DOWN	DOWN
**AT2G33830**	*DRM2*	SALK_054451	N554451	DOWN	DOWN
**AT2G25900**	*TZF1 | ATCTH*	SALK_112158C	N671895	DOWN	DOWN
**AT5G57660**	*COL5*	SALK_096361C	N663414	UP	DOWN
**AT5G56100**	Unknown Protein	SALK_049127C	N681128	DOWN	DOWN
**AT4G36010**	Unknown Protein	SALK_142656C	N679182	UP	UP
**AT5G54960**	*PDC2*	SALK_066678C	N668435	UP	UP
**AT4G23700**	*CHX17*	SALK_063282C	N670826	Not Diff. Expressed	DOWN
**AT5G26220**	*GGCT2*	SALK_056007C	N662534	Not Diff. Expressed	UP
**AT2G39510**	*UMAMIT14*	SALK_037123C	N685665	Not Diff. Expressed	UP
**AT3G03870**	Unknown Protein	SALK_093582C	N655742	DOWN	DOWN
**AT5G66815**	Unknown Protein	SALK_148093C	N667582	UP	UP
**AT1G52700**	Unknown Protein	SALK_031367C	N674312	UP	UP
**AT5G10040**	Unknown Protein	SALK_095117C	N678849	UP	UP
**AT3G63110**	*IPT3*	Kindly provided by Prof. Kakimoto.		UP	UP
**AT3G63110 |AT5G19040 AT3G23630**	*IPT3;5;7*	Kindly provided by Prof. Kakimoto.		IPT3 & 7 : UPs IPT5 not diff. Expressed	IPT3 & 7 : Ups IPT5 not diff. Expressed

Supplemental Table 6: List of genes differentially expressed in the roots during a 22-h LD (this study) and induced by 1-h red light treatment (Molas et al., 2006)

Conditions in which differentially expressed	AGI identifer	Gene name	Present study (FC >2; adjusted p-value ≤ 0,01)		Molas et al., 2006 (FC>2; p value ≤ 0,05)	Same regulation in both experiments?
			Regulation by LD at h16	Regulation by LD at h22	Regulation by red light (RL)	
DEG both at h16, h22, and under red light	AT1G06040	STO	**UP** in LD	**DOWN** in LD	**UP** in RL	NO
	AT5G11260	HY5	**UP** in LD	**UP** in LD	**UP** in RL	**YES**
	AT2G46340	SPA1	**UP** in LD	**UP** in LD	**UP** in RL	**YES**
	AT1G47270	TLP6	**DOWN** in LD	**DOWN** in LD	**DOWN** in RL	**YES**
DEG both at h22 and under red light	AT2G40080	ELF4	/	**UP** in LD	**UP** in RL	**YES**
	AT3G12320	LNK3	/	**DOWN** in LD	**UP** in RL	NO
	AT5G44110	ABCI21	/	**UP** in LD	**UP** in RL	**YES**
	AT3G02910	UNKNOWN	/	**UP** in LD	**UP** in RL	**YES**
	AT5G44920	TIK	/	**DOWN** in LD	**DOWN** in RL	**YES**
	AT1G32690	UNKNOWN	/	**UP** in LD	**UP** in RL	**YES**
	AT5G02270	ABCI20	/	**UP** in LD	**UP** in RL	**YES**

Supplemental Table 7: List of primers used for the RT-qPCR

Gene name	AGI identifier	Primer direction	Sequences (5'-3')	Tm[1]	Amplicon length	References	Computed efficiency[2]	Hybridization temperature
ACT2	AT3G18780	FOR	GGTAACATTGTGCTCAGTGGTGG	61.91	108 bp	D'aloia et al., 2011	88.50%	56°C
		REV	AACGACCTTAATCTTCATGCTGC	59.62				
UBQ10	AT4G05320	FOR	AGAAGTTCAATGTTTCGTTTCATGTAA	58.6	98 bp	Burgos-rivera et al., 2008	89.00%	56°C
		REV	GAACGGAAACATAGTAGAACACTTATTCA	59.94				
GI	*AT1G22770*	FOR	TGGTCCGGCATCAGTATCCATCA	63.42	219 bp	AtRT primer	89.70%	56°C
		REV	CCCATTGCTCCGAATAGTCATTCTC	61.54				
CCA1	*AT2G46830*	FOR	ATCTGGTTATTAAGACTCGGAAGCC	60.45	202 bp	James et al., 2008	93.30%	56°C
		REV	GCCTCTTTCTCTACCTTGGAGAAAA	60.51				
PRR7	*AT5G02810*	FOR	GTCTTTAAGTGCTTATCGAAAGGAGC	60.46	118 bp	James et al., 2008	97.50%	56°C
		REV	CACTACCACTAGAACTTTGGCATCT	60.57				
TPS1	*AT1G78580*	FOR	TGCATCAGTTGATGTTGTCCA	58.14	174 bp	Primer blast	104.80%	56°C
		REV	ACATCTTCGTCCTTCCCCAAG	59.72				
IPT3	*AT3G63110*	FOR	CCGCCGCGAATTACTGTCAC	62.02	175 bp	Primer blast	77.60%	56°C
		REV	AGTGCCACGTCCACCCATAG	61.9				
IPT5	*AT5G19040*	FOR	TCAGGGCCGTCGAGTCAATC	61.95	93 bp	Primer blast	80.40%	56°C
		REV	CGCAATCGTTGACCAGAGCC	61.97				
IPT7	*AT3G23630*	FOR	ATGCATCGTGTTGACGCCAC	61.91	187 bp	Primer blast	79.00%	56°C
		REV	TTCCTCCGCCGTAAGATGCC	62.31				
UNKNOWN	AT3G03870	FOR	CGGACTCTGTTTCGTCACCA	59.97	109 bp	Primer blast	100.20%	56°C
		REV	ATCTGAGGCAGAGGACTCGT	60.03				

[1] Tm were computed using NCBI primer blast with the default parameters.

[2] Efficiencies were computed using a series of five successive 4 X dilutions.

References

D'Aloia, M., Bonhomme, D., Bouché, F., Tamseddak, K., Ormenese, S., Torti, S., Coupland, G., and Périlleux, C. (2011). Cytokinin promotes flowering of Arabidopsis via transcriptional activation of the *FT* paralogue *TSF*. Plant J **65:** 972–979.

Burgos-Rivera, B., Ruzicka, D.R., Deal, R.B., McKinney, E.C., King-Reid, L., and Meagher, R.B. (2008). ACTIN DEPOLYMERIZING FACTOR9 controls development and gene expression in Arabidopsis. Plant Mol Biol **68:** 619–632.

Han, S. and Kim, D. (2006). AtRTPrimer: database for Arabidopsis genome-wide homogeneous and specific RT-PCR primer-pairs. BMC Bioinformatics **7:** 179.

James, A.B., Monreal, J.A., Nimmo, G.A., Kelly, C.L., Herzyk, P., Jenkins, G.I., and Nimmo, H.G. (2008). The Circadian Clock in Arabidopsis Roots Is a Simplified Slave Version of the Clock in Shoots. Science **322:** 1832–1835.

Ye, J., Coulouris, G., Zaretskaya, I., Cutcutache, I., Rozen, S., and Madden, T.L. (2012). Primer-BLAST: A tool to design target-specific primers for polymerase chain reaction. BMC Bioinformatics **13:** 134.

Supplementary Dataset 1

Supplementary Dataset 1 containing root phenotyping data has been permanently deposited on Zenodo and is available at the following address: https://zenodo.org/record/50831. The dataset contains the raw images of the different lines, the tracings of the root system architectures and the scripts used for the analysis.
